# Structural and Electronic Properties of Thiophene-Based Supramolecular Architectures: Influence of the Underlying Metal Surfaces

**DOI:** 10.3390/nano15080572

**Published:** 2025-04-09

**Authors:** Lixia Kang, Yao Tian, Hui Lu, Shunze Xia, Xianfei Xu, Zechao Yang

**Affiliations:** 1 School of Physics, Hangzhou Normal University, No. 2318, Yuhangtang Rd, Hangzhou 311121, China; kanglixia@hznu.edu.cn (L.K.); tianyao@stu.hznu.edu.cn (Y.T.); lu_hui2025@163.com (H.L.); xiashunze@126.com (S.X.); xfxu@stu.hznu.edu.cn (X.X.); 2 Institut für Experimentalphysik, Freie Universität Berlin, Arnimallee 14, 14195 Berlin, Germany

**Keywords:** scanning tunneling microscopy, substrate-mediated self-assembly, molecular orbital alignment, molecular orbital distribution, oligothiophene

## Abstract

Dicyanovinyl (DCV)-substituted oligothiophenes consist of both electron donor and acceptor ligands, which makes them promising materials for organic electronics. Here, we studied the structural and electronic properties of methyl-substituted dicyanovinyl-quinquethiophenes (DCV5T-Me_2_) adsorbed on different metal surfaces, namely Au(111), Ag(111), and Cu(111), by using low-temperature scanning tunneling microscopy/spectroscopy (STM/STS). It is found that the assembled structures of DCV5T-Me_2_ and the corresponding electronic properties vary depending on the underlying substrates. On Au(111) and Ag(111), compact organic islands are formed through intermolecular hydrogen bonding and electrostatic interactions. The lowest unoccupied molecular orbital (LUMO) and LUMO+1 of DCV5T-Me_2_ are lower in energy on Ag(111) than those on Au(111), due to the stronger molecule–surface interaction when adsorbed on Ag(111). Moreover, orbital distributions of the LUMO and LUMO+1 in dI/dV maps on Au(111) and Ag(111) are the same as the DFT-calculated orbital distributions in gas phase, which indicates physisorption. In contrast, chemisorption dominates on Cu(111), where no ordered assemblies of DCV5T-Me_2_ could be formed and resonances from the LUMO and LUMO+1 vanish. The present study highlights the key role of molecule–substrate interactions in determining the properties of organic nanostructures and provides valuable insights for designing next-generation organic electronics.

## 1. Introduction

Molecular electronics, where functional organic molecules are utilized as the elementary building blocks for electronic circuitry, are considered as a potential candidate for next-generation electronics [1,2,3,4,5,6]. Since most of the potential applications of molecular electronics involve low-dimensional organic films growing on top of metal substrates, it is crucial to understand the structural and electronic properties of functional organic molecules when contacting with different types of metal surfaces at atomic or sub-molecular levels [7,8,9]. Benefiting from its ultra-high spatial resolution, scanning tunneling microscopy (STM) is an ideal instrument for microscopically characterizing molecular assemblies on metal surfaces [9]. Lots of effort has been made over the past few decades, and tremendous progress has been achieved in this field [7,9,10]. In general, the supramolecular architectures formed on metal and their electronic properties are mainly mediated by intermolecular interactions and effects from the surfaces, like metal–ligand hybridization, charge transfer, and so on [8,11,12,13,14,15,16,17,18,19,20,21,22,23,24,25,26]. The strength of interactions between molecules and metal surfaces heavily varies depending on the specific system [11,12]. Therefore, it is meaningful to understand for a specific family of functional organic molecules how the molecule–surface interactions influence the bonding nature and corresponding electronic properties by tuning the species of underlying metal substrates.

**Figure 1:**
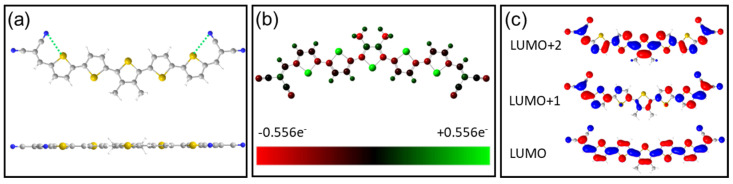
(**a**) The chemical structure and DFT-optimized conformation of the isolated DCV5T-Me_2_ molecule. The color code is white: hydrogen; gray: carbon; blue: nitrogen; and yellow: sulfur. The green dashed lines indicate electrostatic interaction between N and S atoms. (**b**) The calculated charge distribution over a free DCV5T-Me_2_ molecule. The S atoms and N atoms are positively and negatively charged, respectively, resulting in electrostatic interactions between them. (**c**) The calculated orbital structures of DCV5T-Me_2_ with the most stable conformation.

In this work, we studied in particular a derivative of oligothiophene, methyl-substituted dicyanovinyl-quinquethiophenes (DCV5T-Me_2_), by using low-temperature scanning tunneling microscopy/spectroscopy (STM/STS). DCV5T-Me_2_ has an internal electron acceptor–donor–acceptor architecture, where a central electron-rich quinquethiophenes (5T) backbone serves as the donor and two terminal electron-deficient dicyanovinyl (DCV) groups are acceptor (Figure 1a). The internal acceptor–donor–acceptor structure makes DCV5T-Me_2_ an interesting molecule for organic diodes, transistors, and solar cells [27,28,29,30,31,32]. In particular, the introduction of electron acceptor units into oligothiophenes results in reduced band gaps and outstanding photovoltaic properties. Power conversion efficiencies over 10% have been achieved in DCV5T-Me_2_-based heterojunction solar cells [28,29,30,32]. The efficient charge dynamics to a large extent determines the performance of organic electronics composed of DCV5T-Me_2_ molecules and metal substrates, which is strongly influenced by the packing structure, molecular orbital energetic alignment, and spatial distribution [27,33,34,35,36]. The structural and electronic properties of DCV5T-Me_2_ on the Au(111) surface have been intensively studies, where the majority of DCV5T-Me_2_ molecules self-assemble into organic islands and maintain to a large degree their free-molecule character due to relatively weak surface–molecule interaction [33]. On the other hand, a few DCV5T-Me_2_ molecules form metal–organic chains by incorporating Au adatoms from the surface, where the molecular orbital structures are strongly modified by coordination bonding [36]. However, a systematic study on the influence of metal surfaces with different reactivity on the structural and electronic properties of DCV5T-Me_2_ is still missing.

Single crystals of coinage metals, namely Au(111), Ag(111), and Cu(111), were chosen in our study, whose interactions with organic molecules increase, in general, in the order from gold to silver, then to copper. Here, we also observed increasing molecule–surface binding when we moved from Au(111) to Ag(111) to Cu(111). On Au(111), compact islands are formed with all DCV5T-Me_2_ molecules exhibiting the same contrast. DCV5T-Me_2_ also self-assembles into compact islands on Ag(111), while two-thirds of the molecules adopt asymmetric adsorption geometry. DCV5T-Me_2_ on Cu(111), on the other hand, is pinned on the surface avoiding forming compact assemblies. Accordingly, the properties of molecular orbitals are all different for DCV5T-Me_2_ on these three types of noble-metal surfaces. It was found that the spatial distribution of LUMO and LUMO+1 is maintained upon adsorption on Au(111) and Ag(111) surfaces by comparing the DFT calculated orbital structures in gas phase with measured dI/dV maps, indicating physisorption. However, the resonance energies of LUMO and LUMO+1 on Ag(111) are lower around 0.8 eV than those on Au(111), due to relatively stronger metal–ligand hybridization. On Cu(111), the resonance peeks of LUMO and LUMO+1 vanish, which is the result of chemisorption. Moreover, DCV5T-Me_2_ assemblies scatter copper surface electrons, leading to standing waves, due to very strong metal–organic interaction. Our study demonstrated that dicyanovinyl-oligothiophenes exhibit different structural and electronic properties upon adsorption on different metal substrates, which should draw our attention when building corresponding organic electronics.

## 2. Methods

The experiments were performed in a low-temperature STM system working at 4.2 K and under an ultra-high vacuum condition (below 2 × 10^−10^ mbar). The Au(111), Ag(111), and Cu(111) single crystals (from MaTecK, Jülich, Germany) were cleaned by repeated cycles of Ar^+^ sputtering and subsequent annealing to around 800 K. DCV5T-Me_2_ molecules were deposited on the metal surfaces from a home-built molecular evaporator heated to 510 K, which leads to the formation of molecular self-assemblies on the surface. The STM images were measured in the constant current mode and processed with the Nanotec WSxM software (version Beta 9.2) [37]. The dI/dV spectra and maps were acquired with a lock-in amplifier with a typical frequency of 700 Hz and a modulation amplitude of 8 mV.

DFT calculations were carried out by using the GAUSSIAN 03 W program package [38]. The 6-31G basis set and the B3LYP exchange-correlation functional were chosen [27,35,36]. All calculations including the optimization of molecular conformation and molecular orbital alignment and distribution were performed in gas phase. Theoretical studies considering the metal substrates during DFT calculations are necessary to completely understand the mechanisms governing the modification of DCV5T-Me_2_ properties upon adsorption on different metal surfaces.

## 3. Results and Discussion

The DCV5T-Me_2_ molecule has two dicyanovinyl (DCV) functional groups that are symmetrically linked to a central quinquethiophenes (5T) group with the central thiophene ring substituted by two methyl (Me_2_) groups. DFT calculations reveal that there are several types of isomers for DCV5T-Me_2_ in gas phase, with differently oriented thiophene rings and DCV groups. Amongst them, the slightly bent conformation, due to the attachment of methyl groups, with alternately oriented thiophene rings and proximity of cyano groups and sulfur atoms from the neighboring thiophene rings, has the lowest total energy (Figure 1a,b) [36]. The most stable conformation, with a total length of 2.75 nm, adopts C_2v_ symmetry with all the atoms located in the same plane except the hydrogen atoms in the methyl groups. Figure 1c shows the spatial distribution of the LUMO, LUMO+1, and LUMO+2 orbitals for the most stable conformation in gas phase. The LUMO is delocalized over the entire molecule with slightly lower intensity at the DCV groups, while the LUMO+1 is mainly distributed at the two sides and the LUMO+2 is mainly localized at the central part. The calculated orbital spatial distribution in gas phase could be used to compare with the experimentally measured orbital structure to evaluate the influence of the metal surface upon adsorption [8,9].

### 3.1. Assembled Structures and Electronic Properties on Au(111)

**Figure 2:**
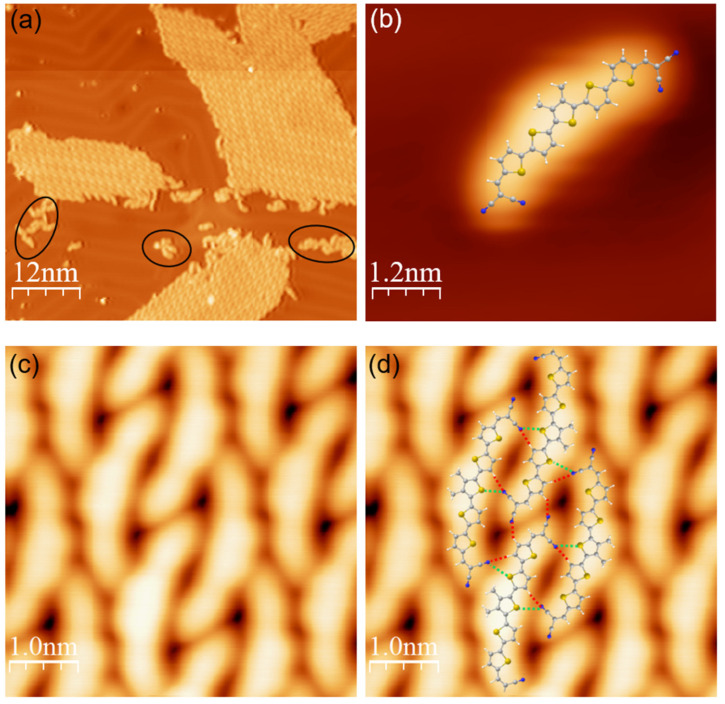
Self-assembly of DCV5T-Me_2_ on Au(111). (**a**) An overview STM image (I = 100 pA, V = 0.8 V) of DCV5T-Me_2_ molecules deposited on room temperature Au(111) surface. The black ovals highlight the clusters and chains. (**b**) High resolution STM image (I = 100 pA, V = 0.3 V) of a single DCV5T-Me_2_ on the surface with the chemical structure model overlaid. (**c**) High resolution STM image (I = 500 pA, V = 0.2 V) of the molecular island. (**d**) The bonding structure of the molecular island. The red dashed lines indicate hydrogen bonds, while the green dashed lines indicate the electrostatic interaction between N and S atoms.

When DCV5T-Me_2_ is deposited on Au(111) at room temperature, it predominantly forms compact islands (Figure 2a). Individual DCV5T-Me_2_ monomers could also be occasionally observed lying on bare gold regions. Figure 2b shows the high resolution STM image of an individual DCV5T-Me_2_, which adopts a bent conformation perfectly fitting with the DFT-optimized geometry in gas phase. High resolution STM of the islands revels that the molecules within the islands have the same conformation as the individual molecules (Figure 2c). Therefore, the DFT-optimized geometry could be used to construct the bonding model of islands. As shown in Figure 2d, the cyano groups of one molecule are pointing to the hydrogen and sulfur atoms of the neighboring molecules, which results in intermolecular hydrogen bonding and electrostatic interactions, thereby forming domains with an extension of hundreds of nano meters in two dimensions. The intermolecular hydrogen bonding between dicyanovinyl-oligothiophenes was imaged at atomic resolution by using low-temperature atomic force microscopy in our previous studies [27,33]. Moreover, the Au(111) surface reconstruction underneath the islands is not perturbed by the adsorption of DCV5T-Me_2_, indicating that the interaction between the gold surface and molecules is relatively weak [39]. It is worth noting that few molecules form small clusters and short chains (highlighted by black ovals in Figure 2a) accompanying the compact islands. Those structure are formed through incorporating Au adatoms by the DCV groups, which was reported in our previous study [36].

**Figure 3:**
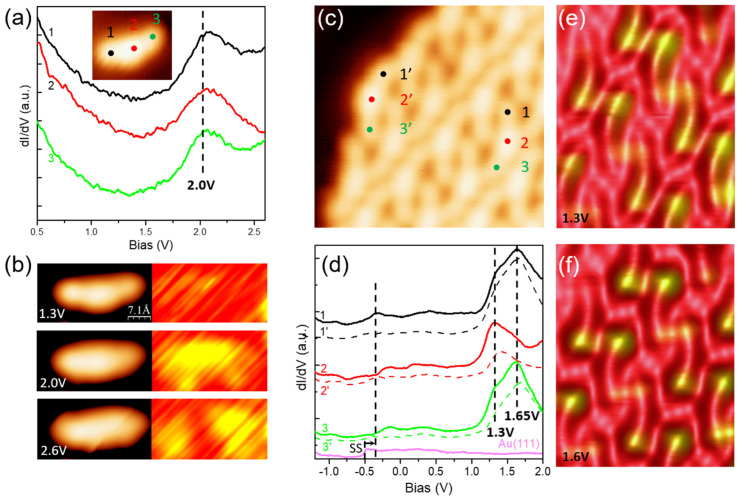
The electronic properties of DCV5T-Me_2_ on Au(111). (**a**) dI/dV spectra acquired with closed feedback (set point: I = 50 pA, V = 0.5 V) at different locations on a single molecule as indicated in the inset STM image. (**b**) The corresponding dI/dV maps of a single molecule recorded at the indicated biases. (**c**) The STM image (I = 100 pA, V = 0.5 V) of a molecular island. (**d**) dI/dV spectra acquired with open feedback (set point: I = 110 pA, V = −2 V) at different locations on the island as indicated in (**c**). The surface state is shifted up around 0.2 V due to the change in work function by the attachment of the organic layer. (**e**,**f**) The corresponding dI/dV maps of a molecular island recorded at the indicated biases. In the maps, the red color represents the background, while the yellow stands for the dI/dV signal.

STS was utilized to investigate the electronic structure of organic islands. As a reference, we measured first the spectra on an individual DCV5T-Me_2_ molecule. Differential conductance spectra recorded on the center and on the DCV end-groups of a DCV5T-Me_2_ molecule show a clear peak at 2.0 V (Figure 3a) with similar intensity at the three locations, which is assigned to the LUMO-derived resonance. In contrast, the LUMO+1 with an onset of 2.5 V is only distributed at the two ends. The extended and local character of the LUMO and the LUMO+1, respectively, are further confirmed by the dI/dV maps acquired at 2.0 V and 2.6 V (Figure 3b). The spatial distribution of LUMO and LUMO+1 on Au(111) is in good agreement with the molecular orbital shape obtained from our DFT calculations in gas phase (Figure 1c), which demonstrates that DCV5T-Me_2_ molecules maintain to a large degree their free-molecule character upon adsorption on Au(111).

STS spectra in the molecular island (Figure 3c,d) show a resonance at 1.3 V distributed over the whole molecule, and a higher-lying resonance at 1.65 V, which is mainly localized at the molecular sides. This distribution is further confirmed by dI/dV mapping, as shown in Figure 3e,f. By comparing these results with the distribution of states over an individual molecule, we assign these two resonances to states derived from the LUMO and LUMO+1 of the molecule within the islands, respectively.

These results show that the LUMO and LUMO+1 are shifted down to 1.3 V and 1.65 V in the islands from 2.0 V and >2.5 V at the single molecule, respectively, on Au(111). Spectra recorded on the molecule at the edge of the island (dash curves in Figure 3d) exhibit states with similar energies with those at the molecule within the island (solid curves in Figure 3d). It indicates that the screening effect does not strongly influence the resonance alignment here. Therefore, we attribute this shift to the presence of intermolecular interactions, which is the only possible origin for the change in electronic properties. Studies have demonstrated that the LUMO and the LUMO+1 states of PTCDA molecules within different domains, where the average hydrogenbonds length varies 0.3 Å, are different in energy. The unoccupied molecular orbitals in the domain with stronger hydrogen bonds are shifted down 0.35 V overall with respect to the states in the domain with weaker bonding. Similarly, studies on BDATB, PTCDI, and BDATB/PTCDI assemblies reveal that the intermolecular hydrogen bonds may create dipoles within the bonding regime, thereby inducing a shift in the molecular resonances in energy [40,41]. Based on these studies, we propose that the downshift in the LUMO and LUMO+1 of DCV5T-Me_2_ within the organic islands originates from the intermolecular hydrogen bonds and electrostatic interaction [27]. Here, the value of the shift is around 0.4 V, which is two times bigger than the reported value (0.35 V). However, it is reasonable considering that, in our system, the transition is from the absence of bonding to the presence of a big amount of strong intermolecular interactions rather than from weaker bonding to stronger bonding.

We can use the quantum mechanism of “particle in a box” to explain the downshift of molecular orbital energy [42]. Strong intermolecular interactions benefit the orbital overlap between molecules. Consequently, the distribution of electrons is more delocalized over the two dimensional domain than within one single molecule. The bigger the size of the box, the lower the energy of the states. Therefore, islands possess orbitals with lower energies than a single molecule in our system.

A closer look at the spectra in Figure 3d reveals small step-like peaks around −0.3 V, which originated from the gold surface state. The surface state penetrates the organic layer and is shifted from −0.5 eV up to −0.3 eV, caused by the change in work function due to the adsorption of organic molecules. This further demonstrates the physisorption and weak molecule–surface interaction on Au(111).

### 3.2. Assembled Structures and Electronic Properties on Ag(111)

**Figure 4:**
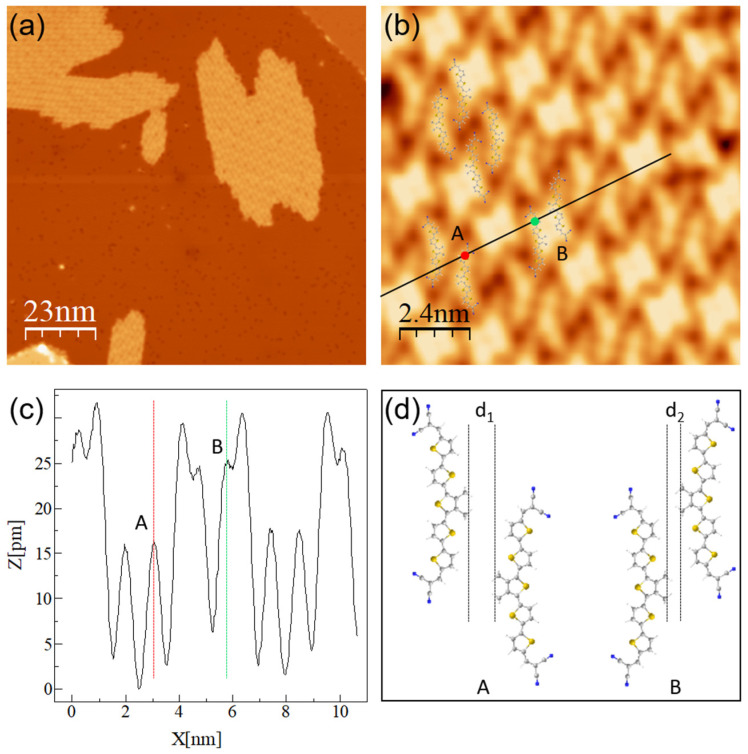
Self-assembly of DCV5T-Me_2_ on Ag(111). (**a**) Overview STM image of DCV5T-Me_2_ islands (I = 15 pA, V = 0.78 V). (**b**) High resolution STM images (I = 70 pA, V = 0.79 V) of island revealing two types of molecules with different height contrasts. (**c**) Line profile acquired across black line in image (**b**). (**d**) Models showing distances between neighboring molecules in images in (**b**). d_1_ = 0.43 nm, d_2_ = 0.22 nm.

The properties of DCV5T-Me_2_ on Ag(111) are expected to be different from the features observed on the relatively inert Au(111) surface. First of all, the work function is different for these two substrates, which will simply shift the orbital alignment in energy [43,44]. Secondly, the strength of hybridization between metal surfaces and organic molecules could modify molecular geometry. Indeed, the adsorption of DCV5T-Me_2_ on Ag(111) shows a different result from that on Au(111). As shown in Figure 4a, large islands of DCV5T-Me_2_ are also formed on Ag(111), on the other hand, without accompanying metal–organic structures (like short chains on Au(111)). Moreover, the high resolution STM image in Figure 4b reveals that there are two types of molecules with different height contrast upon packing in islands. One type exhibits homogenous contrast like on Au(111), named type A. In contrary, for the other type, one side and the center of the molecule are imaged brighter than the other side, which are labeled as type B. The brighter part of the type B molecule is 10 pm higher than type A (Figure 4c). Moreover, type B molecules arise in pairs and the brighter contrast is localized at their contacting regions (model in Figure 4b). Type A molecules emerge in pairs as well, however, with a longer intermolecular distance (model Figure 4b). The intermolecular distance between type A molecules is measured as 0.43 nm, which is 0.22 nm for the type B molecules (Figure 4d). Therefore, we attribute the higher apparent height of type B molecules to the closer intermolecular distance, which probably forces molecules to point their methyl groups outwards from the surface.

**Figure 5:**
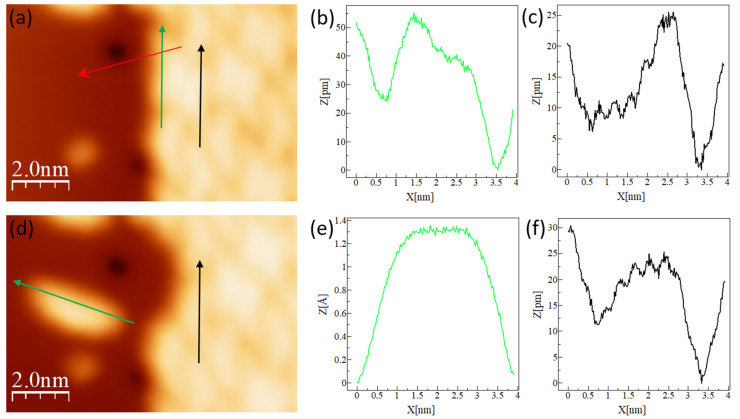
Contrast changes by lateral manipulation. (**a**,**d**) STM images (I = 20 pA, V = 0.76 V) before and after extracting a molecule from a compact domain (set point of the lateral manipulation: I = 100 nA, V = 0.01 V). The movement trace of the tip during manipulation is indicated by the red arrow in (**a**). An intact single molecule is obtained after the manipulation. (**b**,**c**) Line profiles show the height of two type B molecules before the manipulation. (**e**,**f**) Line profiles show the height of the same molecules after the manipulation. The sides with arrows have bigger values in the X axis in the profiles.

Lateral manipulation further demonstrates the dense packing origin of the intra-molecular contrast [24,45,46,47]. After removing its neighbor, a type B DCV5T-Me_2_ molecule is transformed to the type A molecule, whose two sides adsorb with the same height (Figure 5c,f). The same change happened to the resultant single molecule after dragging it out of the island (Figure 5b,e). If we ignore the contrast of individual molecules, it is found that the basic packing pattern of islands on Ag(111) and those on Au(111) is the same (compare models in Figure 4b with Figure 2d). Hence, we propose that intermolecular hydrogen bonds and electrostatic interaction are also responsible for the formation of DCV5T-Me_2_ islands on Ag(111).

**Figure 6:**
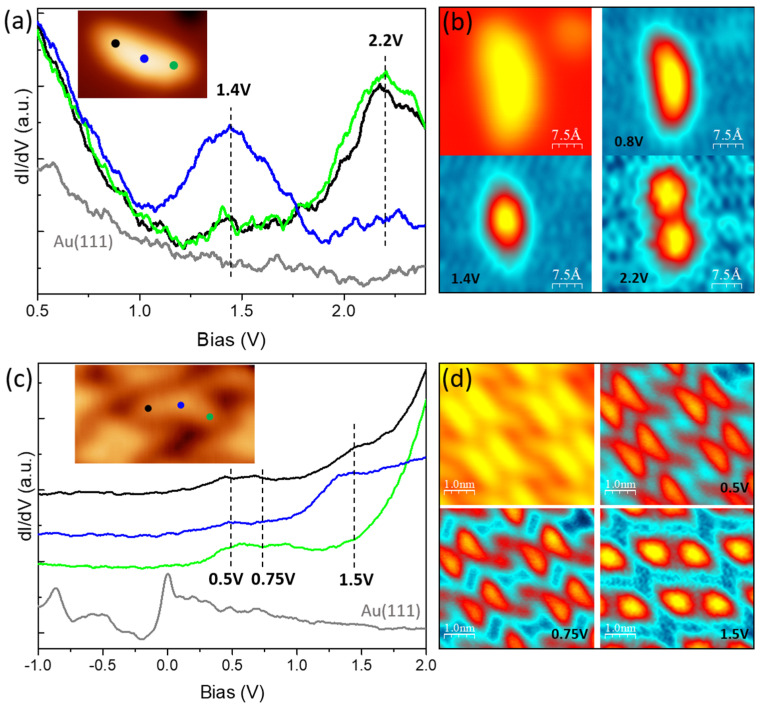
Electronic properties of DCV5T-Me_2_ on Ag(111). (**a**) dI/dV spectra acquired with closed feedback (set point: I = 8 pA, V = 2.4 V) at different locations on a single molecule as indicated in the inset STM image. (**b**) Constant current STM images (left upper panel: I = 20 pA, V = 0.76 V) and corresponding constant height dI/dV maps (the other three panels) recorded at the indicated biases. (**c**) dI/dV spectra acquired with open feedback (set point: I = 50 pA, V = 2.0 V) at different locations of the molecule in an island as the indicated inset (I = 16 pA, V = 0.8 V). Energetic positions of the resonances in the dI/dV spectra are highlighted by black dashed, vertical lines. All the spectra are offset for clarity. (**d**) Constant current STM image (left upper panel: I = 14 pA, V = 0.56 V) and corresponding constant height dI/dV maps (the other three panels) recorded at the indicated biases.

STS measurements were performed to inspect the electronic properties of DCV5T-Me_2_ on Ag(111). The single molecule is a good reference to study the evolution of electronic properties of DCV5T-Me_2_ upon self-assembly into islands. dI/dV spectra (Figure 6a) show that the LUMO at 1.4 V is mainly distributed at the central part of the single molecule with a lower weight at the two sides, whereas the LUMO+1 at 2.2 V is mainly localized at the two sides. This distribution is further imaged in the dI/dV maps shown in Figure 6b and fits with the DFT-calculated orbital structure of DCV5T-Me_2_ in gas phase (Figure 1c).

The orbital energy alignment is changed upon self-assembly on the Ag(111) surface. Figure 6c shows spectra recorded over a type A molecule within an island. Here, three resonances are observed. The state at 0.5 V is distributed over the whole molecule, while the resonance at 0.75 V is mainly located at the two sides. By comparing the type A molecule with the distribution of unoccupied molecular orbitals over the single molecule (Figure 6a), we assign these two states to the LUMO and LUMO+1. The downshift in energy with respect to the single molecule is ascribed to intermolecular interactions, the same mechanism as the case on Au(111) [40,41]. In addition, the center possesses a higher-lying state at 1.5 V. Since DFT calculations show the LUMO+2 located at the center of DCV5T-Me_2_ (Figure 1c), this 1.5V resonance can be assigned to the down-shifted LUMO+2 after assembly. The type B molecule exhibits the same features, which can be demonstrated through dI/dV mapping (Figure 6d). The map acquired at 0.5 V shows similar contrast with the topography due to the delocalization of the LUMO, while signals are located at the two sides and the center at maps taken at 0.75 V and 1.5 V corresponding to the LUMO+1 and LUMO+2, respectively.

Qualitatively, the behaviors of molecular islands on Ag(111) are the same as on Au(111). However, the exact values of orbital energies differ by 0.6 ± 0.2 V. The difference in quantity can be interpreted by considering the work function of surfaces. The work function of Ag(111) is lower by 0.57 V than that of Au(111) [43], which is in good agreement with the down-shifted values of orbital energies. It means that organic islands physically adsorb on both substrates without significant hybridization at the interfaces, resulting in only a rigid shift in orbital energetic alignment [40,41].

### 3.3. Assembled Structures and Electronic Properties of Cu(111)

**Figure 7:**
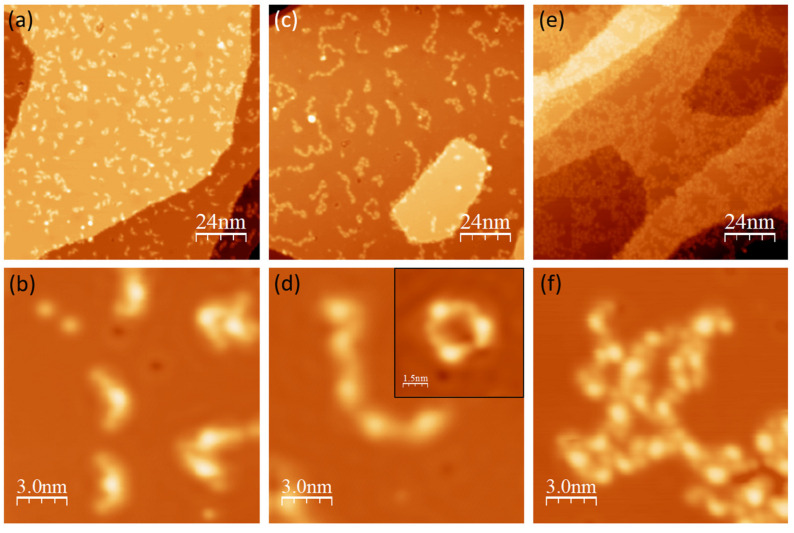
Adsorption and assembly of DCV5T-Me_2_ on Cu(111). (**a**,**c**,**e**) Overview STM images (I = 23, V = 0.33 V for a; I = 12 pA, V = 0.1 V for c; I = 100 pA, V = 0.62 V for e) of deposition of DCV5T-Me_2_ on sample with different temperatures, 180 K, 230 K, and 300K, respectively. (**b**,**d**,**f**) Corresponding high resolution STM images (I = 7.7 pA, V = 0.32 V for b; I = 8.1 pA, V = 0.1 V for d; I = 210 pA, V = 0.74 V for f). Inset in b shows pore (I = 14 pA, V = 0.11 V) consisting of three molecules.

The free molecular properties are not strongly modified by both the Au(111) and Ag(111) substrates. However, a copper surface is expected to induce drastic disturbance to organic materials through strong metal–ligand hybridization. First of all, it is found that the self-assembly of DCV5T-Me_2_ on Cu(111) strongly depends on the substrate’s temperature. At low temperature (180 K), single molecules or small clusters randomly disperse on the surface, as shown in Figure 7a. The high resolution STM image in Figure 7b shows that molecules adopt various conformations, due to the lack of thermal-activated relaxation upon adsorption. Moreover, the two sides exhibit much lower apparent height than the central part. A reasonable explanation is that the end DCV groups and the thiophene backbone strongly interact with the surface, pulling the two methyl groups in the center ring upward. Molecules assemble into chains and pores when deposited on the substrate with higher temperature (230 K), as shown in Figure 7c. In these structures, adjacent molecules connect with each other via their end cyano moieties (Figure 7d). Such a bonding conformation is highly unstable for the bare electronegative nitrogen terminations, unless a metal atom bridges them in between [23,36,48,49,50]. Therefore, we propose that Cu adatoms are incorporated for bridging the electronegative end cyano groups. Differently from pure organic islands on Ag(111), the formation of pure metal–organic motifs on Cu(111) means that the interaction between Cu adatoms and cyano groups is much stronger than the intermolecular interactions. Disordered clusters are formed when depositing molecules on a room temperature sample (300 K), as shown in Figure 7e. The high resolution STM image in Figure 7f reveals that most of the molecules are not intact. The decomposition can be attributed to bond dissociation probably catalyzed by Cu adatoms on the Cu surface [51].

**Figure 8:**
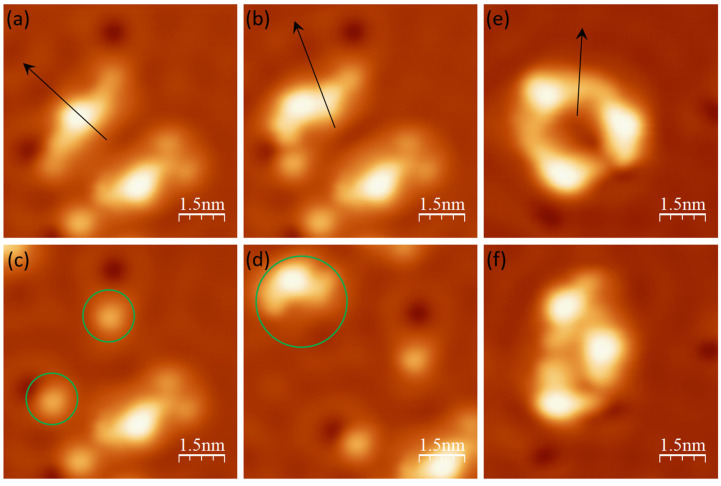
Lateral manipulation. (**a**–**c**) STM images (I = 100 pA, V = 0.1 V) of a lateral manipulation sequence of a single DCV5T-Me_2_ molecule on Cu(111). (**d**) STM image (I = 100 pA, V = 0.1 V) of the dissociated molecule after manipulation. The green circles in (**c**,**d**) highlight the DCV groups and the backbone of the molecule, respectively. (**e**,**f**) STM images (I = 14 pA, V = 0.11 V) before and after a lateral manipulation of a three-molecule pore. During lateral manipulation, the movement trace of the tip is indicated by the black arrows in the STM images.

Lateral manipulations were performed to obtain more insight into the adsorption and assembly behaviors of DCV5T-Me_2_ on Cu(111). Figure 8a–c shows a lateral manipulation sequence of a single molecule on a cold sample (180 K). One can identify that, after manipulation, two small dots stay at their original positions, while the molecular backbone (Figure 8d) is moved away by the tip. It demonstrates that the end cyano groups are pinned at the surface upon adsorption due to strong interaction with the Cu atoms underneath. The interaction is even stronger than the covalent bonds within DCV5T-Me_2_, thereby dissociating the molecule during lateral manipulation. In contrast, lateral manipulation on a pore consisting of three molecules shows different phenomenon. The whole structure is dragged to another position by the tip. Although conformations are changed, molecules remain intact and are still connected via their ends. This further certifies the incorporation of Cu adatoms by ligands due to thermal activation at a moderate temperature (230 K) sample. As a result, lateral coordination bonding dominates over vertical interactions, which is so robust that the metal–organic motif can be laterally manipulated as a whole without dissociation. Note that the same phenomenon happens when manipulating the chain, indicating its combination of molecules with Cu adatoms.

**Figure 9:**
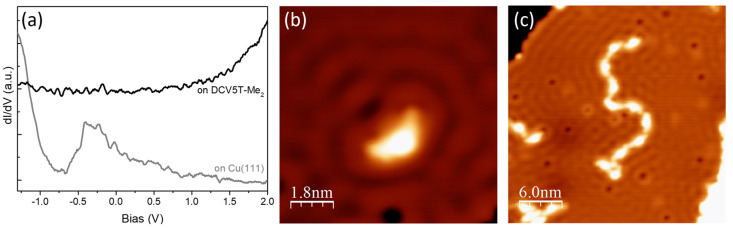
(**a**) dI/dV spectra acquired with open feedback (set point: I = 20 pA, V = 2.0 V) at DCV5T-Me_2_ showing no feature from −1.3 V to +2.0 V. (**b**,**c**) STM images (I = 16 pA, V = 0.14 V for (**a**); I = 74, V = 0.1 V for (**b**)) of standing waves formed through scattering surface electrons by single molecule and chain, respectively.

We performed STS measurements at DCV5T-Me_2_ on Cu(111) to inspect its electronic structure. In contrast to the rich spectral information obtained when adsorbed on Au(111) and Ag(111), there is no feature within the bias range from −1.3 V to +2.0 V (Figure 9a) due to the strong hybridization with Cu surface [12]. Note that the absence of resonances is ubiquitous for spectra recorded at molecules in different assemblies and different positions of the same molecule.

In general, the strong interaction between molecules and substrates enables scattering of surface electrons [8,52,53]. Figure 9b shows standing waves surrounding a single DCV5T-Me_2_ molecule. The oscillation period is around 15 Å, corresponding to half of the Fermi wavelength (λ_F_) of the Cu(111) surface. Similarly, the metal–organic chains can also serve as a scattering center, as shown in Figure 9c. This behavior is absent on Au(111) and Ag(111) because of relatively weaker interactions between the surfaces and ligands.

**Figure 10:**
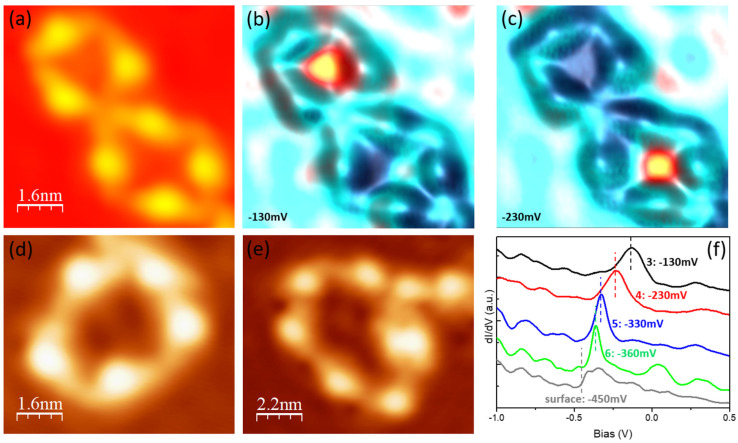
The confinement of the surface electrons in the metal–organic pores. (**a**) An STM image of two pores consisting of three and four molecules (I = 80 pA, V = −0.23 V). (**b**,**c**) Corresponding constant current dI/dV maps recorded at the indicated bias voltages. The dI/dV signals (yellow and red) are superimposed over the topography (cyan). (**d**,**e**) STM images of two pores consisting of five and six molecules, respectively. (**f**) dI/dV spectra acquired with open feedback (set point: I = 80 pA, V = 1.0 V) at the center of pores consisting of a different number of molecules, from three to six. The energetic positions of the resonances in the dI/dV spectra are highlighted by the dashed vertical lines.

Consequently, surface electrons will be confined within the corresponding sealed structures, for example, the metal–organic pores [8]. As shown in Figure 10a–c, electrons are localized at pores consisting of three and four molecules, respectively. Pores with different sizes have different eigenstates. dI/dV spectra were recorded at the center of pores, with sizes ranging from three to six molecules (Figure 10a,d,e), to inspect the energy shift in confined surface electrons. The first eigenstate is monotonously shifted down from −130 meV to −360 meV as the pore size increases, as shown in Figure 10f. This phenomenon can be interpreted by the quantum mechanism of a particle in a two dimensional circular box [54,55]. The eigenstate of order (*m*, *n_r_* + 1) of electron gas confined in a box, can be described as follows:

(1) $$E=\left[\frac{\hbar^{2}}{2m_{e}R^{2}}\right]{\left[a_{\left(m,n_{r}\right)}\right]}^{2}+E_{s}$$

where *m_e_* is the electron effective mass, *R* is the radius of the circle, $a_{\left(m,n_{r}\right)}\;$ can be calculated from the Bessel function, and *E_s_* is the natural onset of the surface state (−450 meV for Cu(111)). Therefore, in energy, the ground state of confined electrons is gradually shifted down to the surface state from the pore with a smaller radius to that with a bigger radius, which is associated with a weaker confinement effect. The self-assembly of different sizes of sealed nanostructures on metal surfaces offers a way to fabricate tunable surface electron states.

## 4. Conclusions

DCV5T-Me_2_ exhibits different self-assembly behaviors on Au(111), Ag(111), and Cu(111) as a result of the competition of lateral interactions between molecules, and between molecules and adatoms on surfaces. Pure organic islands are self-assembled on the Au(111) and Ag(111) substrates due to strong intermolecular attractive interactions. Differently, molecule/Cu-adatom coordination dominates the structure formation when deposited on Cu(111), inducing homogenous metal–organic motifs on the surface at room temperature. Vertical interactions between molecules and surfaces vary as well. DCV5T-Me_2_ physically adsorbs on Au(111) and Ag(111), while chemisorption occurs on Cu(111), resulting in scattering and confinement of surface electrons. Molecular electronic properties evolve accordingly. Molecular orbital energetic alignment is rigidly shifted down on Ag(111) with respect to that on Au(111) due to lower work function. In contrast, molecular resonances vanish on Cu(111) owing to strong organic/metal hybridization. The changes in structural and electronic properties of DCV5T-Me_2_ assemblies on different metal substrates should draw our attention when building corresponding organic electronics.

## Data Availability

Data are contained within the article.
